# Malignant Melanoma in a Retrospective Cohort of Immunocompromised Patients: A Statistical and Pathologic Analysis

**DOI:** 10.3390/cancers15143600

**Published:** 2023-07-13

**Authors:** Trevor F. Killeen, Ryan Shanley, Vidhyalakshmi Ramesh, Alessio Giubellino

**Affiliations:** 1 Department of Laboratory Medicine and Pathology, University of Minnesota, Minneapolis, MN 55455, USA; 2 Masonic Cancer Center—Biostatistics Core, University of Minnesota, Minneapolis, MN 55455, USA; 3 Masonic Cancer Center—Clinical Informatics Shared Services, University of Minnesota, Minneapolis, MN 55455, USA; 4 Masonic Cancer Center, University of Minnesota, Minneapolis, MN 55455, USA

**Keywords:** melanoma, immunosuppression, immunocompromised, solid organ transplant, bone marrow transplant

## Abstract

**Simple Summary:**

Despite recent advances in immunotherapy, melanoma remains the deadliest cutaneous malignancy. Immunocompromised patients are at heightened risk of developing melanoma and experience greater levels of morbidity and mortality compared to their non-immunocompromised peers with the disease. We sought to characterize immunocompromised patients with melanoma at our institution in terms of histopathologic parameters, trends in survival analysis, and comparison to the general population using the Surveillance, Epidemiology, and End Results (SEER) database. Analysis of our transplant sub-cohort revealed an overall melanoma standardized incidence ratio of 1.53, though variation was seen based on transplant type, sex, and time since transplantation. Our study reveals important trends in disease among immunocompromised patients with melanoma at an academic tertiary-care center and is valuable for clinicians caring for this unique demographic of patients.

**Abstract:**

Background: Malignant melanoma is the leading cause of death due to cutaneous malignancy. Immunocompromised individuals have an elevated risk of developing melanoma. We aimed to provide histopathologic and statistical characterization of melanoma development in immunocompromised patients. Methods: We reviewed our institution’s databases to identify all patients with a confirmed history of immunosuppression who subsequently developed melanoma, focusing on diagnoses during the follow-up period of 2011–2019. A total of 93 patients with a combined 111 melanoma lesions were identified. Results: Common causes of immunosuppression included transplantation and lymphoproliferative disorders. Superficial spreading and lentigo malignant melanoma were the most common malignant melanoma subtypes. Median Breslow depth was 0.7 mm, and the most common primary tumor stage was T1a. Our transplant sub-cohort had an overall melanoma incidence of 0.9 per 1000 person-years (95% CI 0.66 to 1.20) and a standardized incidence ratio (SIR) of 1.53 (95% CI 1.12 to 2.04) relative to a general population cohort from the Surveillance, Epidemiology, and End Results Program (SEER). Conclusions: We report histopathologic characteristics of immunocompromised patients developing melanoma at a large academic tertiary-care center. Differences in age, sex, time since transplantation, and transplant type may play a significant role in melanoma SIR in this patient demographic.

## 1. Introduction

Cutaneous malignancies represent the most common forms of cancer [1,2]. Although representing only about 1% of cutaneous malignancies, melanoma is the leading cause of skin cancer-related death [3]. Following many years of increase, there has been a recent stabilizing of melanoma incidence, though, in 2022, it was estimated that nearly 100,000 Americans would be diagnosed with melanoma and over 7500 would die of the disease [4]. These data reflect a recent reduction in melanoma-related mortality due to the introduction of more efficacious therapeutic treatments, such as targeted therapy with BRAF and MEK inhibitors and the use of immunotherapy as the new standard of care for advanced melanoma stages [5,6].

There are several well-recognized risk factors for melanoma, including ultraviolet (UV) light exposure, genetic predisposition, and specific demographic or clinical characteristics [3,7,8]. In particular, immunocompromised individuals have an elevated risk of developing all cutaneous malignancies, including melanoma [9]. Moreover, these immunocompromised patients tend to have more aggressive and advanced disease, as previously reported [10], compounding on traditional pathologic prognostic factors such as Breslow depth and ulceration.

The relatively recent success of immunotherapy for high-stage melanoma demonstrates the role of the tumor microenvironment and the immune system in disease progression and the unique challenges posed by melanoma in immunocompromised patients. An increased risk of developing melanoma has been reported in several categories of immunocompromised patients, including solid organ transplant recipients, leukemia and lymphoma patients, and HIV/AIDS patients [11]. The relative causes of immunosuppression in these patients cover a broad range of etiologies. Immunosuppression in solid organ transplant patients is attributed to iatrogenic medications to prevent organ rejection, including a combination of antiproliferative agents, calcineurin inhibitors, corticosteroids, mammalian target of rapamycin (mTOR) inhibitors, and T cell co-stimulation blockers; transplant patients remain on a maintenance regimen of immunosuppressive medication, following initial induction therapy, for many years, thus increasing the risk of several secondary diseases, including malignancies [12].

In other categories of immunosuppression, such as hematologic malignancies and lymphoproliferative diseases (leukemias, lymphomas, and multiple myeloma), immune cell dysfunction may be attributed to the malignancy itself, as well as the bone marrow or stem cell transplantations and immunosuppressive chemotherapy that patients may receive [11].

In patients infected with HIV who develop AIDS, chronic immunosuppression is attributed primarily to CD4+ helper T cell depletion [13,14]. Current antiretroviral triple therapy regimens in the post-HAART era prolong the life of these patients to match that of the general population, but this trend has also uncovered an increased risk of developing non-HIV-related malignancies, including malignant melanoma [15,16].

In the present study, we performed a retrospective analysis of melanoma in immunocompromised patients from our institution during a follow-up period of almost ten years. We reviewed both clinical and histopathologic information of our cohort, and we estimated overall and melanoma-specific survival.

## 2. Materials and Methods

### 2.1. Cohort Data

This study received IRB approval from our institution. We reviewed our institutional database to identify all patients with a history of immunosuppression who developed melanoma between 2011 and 2019 using both ICD-10 codes and natural language search parameters. Following manual verification and chart review, 93 qualified for inclusion by these criteria. Although manual review revealed some patients with a melanoma diagnosis prior to 2011 who were incorporated into the study for histopathologic data, only those patients with an initial diagnosis during the 2011–2019 follow-up period were incorporated into our SEER analysis (see below).

Full clinical and histopathologic data were obtained for the final list of 93 patients. Among the 93 patients, there were a total of 111 unique melanoma lesions. For all patients, we were able to obtain the following clinical data: gender; date of and age at onset of immunosuppression; date of and age at melanoma diagnosis; time between onset of immunosuppression and melanoma diagnosis (initial melanoma diagnosis in those with multiple lifetime melanoma lesions); and cause of immunosuppression. Allogeneic bone marrow or stem cell transplantation was considered a primary cause of immunosuppression when performed shortly after diagnosis and as therapy for lymphoproliferative disorders (e.g., multiple myeloma). In cases of patients with lymphoproliferative disorders who did not undergo allogeneic bone marrow or stem cell transplantation, immunosuppression was attributed to the lymphoproliferative disorder itself. Autologous bone marrow or stem cell transplantation was not considered a primary cause of immunosuppression in any patients but was recorded and incorporated into our SEER analysis (see below). We refer to leukemia, lymphoma, monoclonal gammopathy of undetermined significance, and multiple myeloma collectively as lymphoproliferative disorders (LPD). Note also that “bone marrow” and “stem cell” transplantations are often collectively simply called “bone marrow transplantation” (BMT), particularly in the analysis of our institution’s transplant database records.

We reviewed our medical records and our state’s death records to identify all deceased patients in the cohort. When available for deceased patients, we obtained both the cause and manner of death. The date of and age at death, as well as the time between the onset of immunosuppression and death and the time between melanoma diagnosis and death, were recorded. For all living patients, the number of follow-up years since the melanoma diagnosis was also recorded.

### 2.2. Solid Organ Transplant and Lymphoproliferative Disorder Population

For all patients with immunosuppression attributed to solid organ transplantation (SOT), organ type was recorded. Likewise, when available, for patients with immunosuppression attributed to leukemia or lymphoma, the specific subtype was recorded.

### 2.3. Lesion Location

The melanoma-specific location on the body, as indicated in Epic or Copath records, was recorded and generalized to the following regions: head and neck, lower extremities, ocular, trunk, and upper extremities.

### 2.4. Histopathology Data

Histopathologic data were obtained through both Epic and Copath records. When available, all the following histopathologic parameters were obtained: melanoma histologic subtype (as defined by the 8th Edition of the American Joint Committee on Cancer (AJCC) melanoma staging system); Breslow depth (mm); Clark level; and others (Table 1).

### 2.5. Statistical Analysis

We used Kaplan–Meier estimates for survival calculations from the date of melanoma diagnosis. Survival and follow-up times for each patient were rounded down to the nearest integer year. For patients who had BMT or SOT with a melanoma diagnosis recorded during the follow-up period, melanoma incidence was calculated by dividing the number of incident cases by the total number of post-transplant follow-up years. Follow-up years included the time a patient was known to be alive without a prior melanoma diagnosis between 2011 through 2019 (the same time period that was used to identify incident cases). We calculated confidence intervals for incidence and incidence ratios by assuming that the number of cases followed a Poisson distribution [17].

### 2.6. SEER Database Search

We used data from the Surveillance, Epidemiology, and End Results (SEER) program of the U.S. National Cancer Institute (NCI) to estimate the incidence of melanoma by age and sex in the non-Hispanic white U.S. population (all patients in our transplant cohort with a recorded racial/ethnic group were non-Hispanic white). The SEER-21 database is a cancer incidence registry that covers 21 geographically diverse areas of the U.S. We used results from 2014 to 2018, which included age categories in five-year increments [18]. Age and sex-standardized incidence ratios (SIRs) were calculated as follows: each study participant’s expected annual probability of melanoma was estimated using the incidence in the age and sex-matched SEER-21 population. The sum of these probabilities over all study participants and follow-up years was the expected number of melanoma cases in the entire cohort based on the reference population. The SIR was the ratio of observed to expected melanoma cases.

## 3. Results

### 3.1. Clinical Characteristics

A total of 93 individual patients with a combined total of 111 unique melanoma lesions were identified from our institutional database. Of these patients, 65 were male, and 28 were female. At the time of analysis, 34 of 93 patients were deceased. The most common causes of immunosuppression pre-dating a melanoma diagnosis included SOT (n = 37), LPD (leukemia, lymphoma, monoclonal gammopathy of undetermined significance, or multiple myeloma) (n = 39), and BMT (n = 10) (Table 1). Among the 37 SOT patients, kidney transplantation was the most common (n = 20) (Table 1). All patients with leukemia (n = 9) had chronic lymphocytic leukemia (CLL). Lymphoma subtypes were variable in electronic health record descriptions and included anaplastic large cell, Burkitt, cutaneous T cell, diffuse large B cell, Hodgkin, malignant B cell, mantle cell, non-Hodgkin, small B cell, and T cell lymphomas.

Lesion body location data were available for 107 melanoma lesions and can be seen in Table 1.

**Figure 1:**
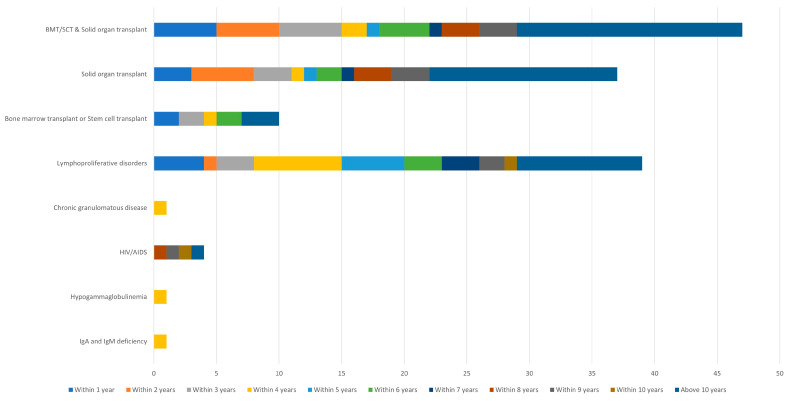
Years from onset of immunosuppression to diagnosis of melanoma. Bar graph representation of time to onset of melanoma from initial diagnosis of a condition leading to patient’s immunosuppression status. Abbreviations: BMT/SCT, bone marrow transplant/stem cell transplant.

For well-represented immunosuppressive categories (n ≥ 10 patients), the median time from immunosuppressive onset to initial melanoma diagnosis can be seen in Table 1 and Figure 1, acknowledging that follow-up time is a confounding variable that impairs clinically meaningful comparisons of lag time across immunosuppressive categories.

### 3.2. Histologic Characteristics

General histologic subtype (invasive vs. in situ melanoma) was available for 101 lesions; 68 lesions were invasive melanomas, while 33 were melanomas in situ. Melanoma subtypes were available for 57 lesions and can be seen in Table 1.

AJCC primary tumor classification was available for 41 lesions (Table 1).

**Figure 2:**
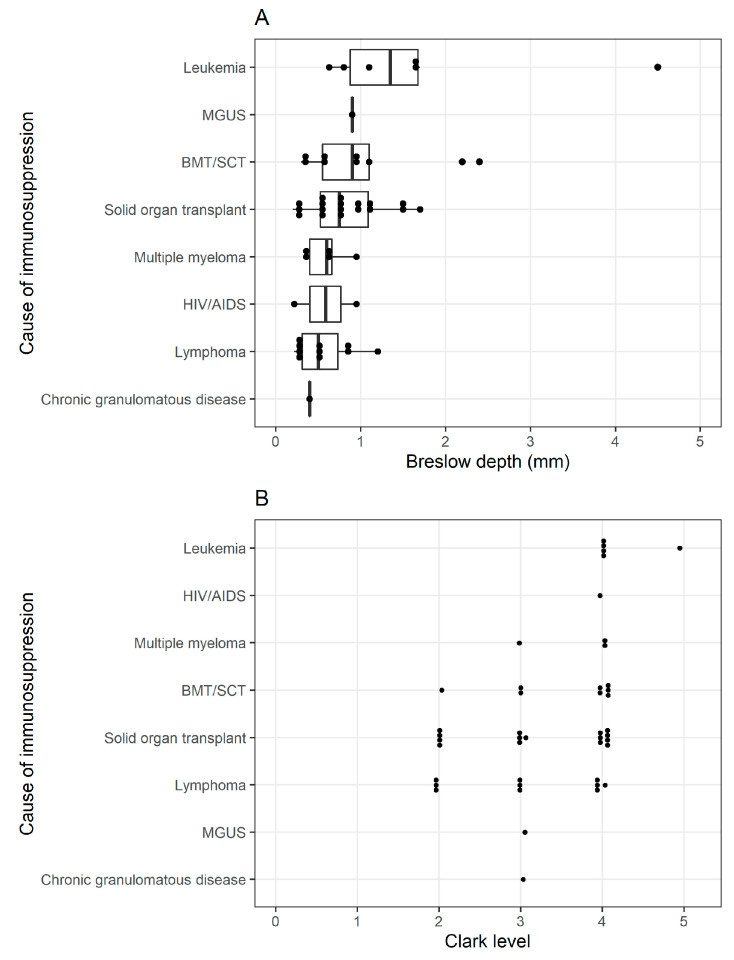
Histopathologic data. (**A**): Dot plot of Breslow depth of melanoma lesions by cause of immunosuppression. Abbreviations: BMT/SCT, bone marrow transplant/stem cell transplant; MGUS, monoclonal gammopathy of undetermined significance. (**B**): Dot plot of Clark level of melanoma lesions by cause of immunosuppression. Abbreviations: BMT/SCT, bone marrow transplant/stem cell transplant; MGUS, monoclonal gammopathy of undetermined significance.

Breslow depth was available for 52 lesions, with a median value of 0.70 mm and subdivided by cause of immunosuppression in Figure 2A.

Clark level was available for forty-four lesions with a median value of four and subdivided by cause of immunosuppression in Figure 2B.

### 3.3. Survival Analysis

**Figure 3:**
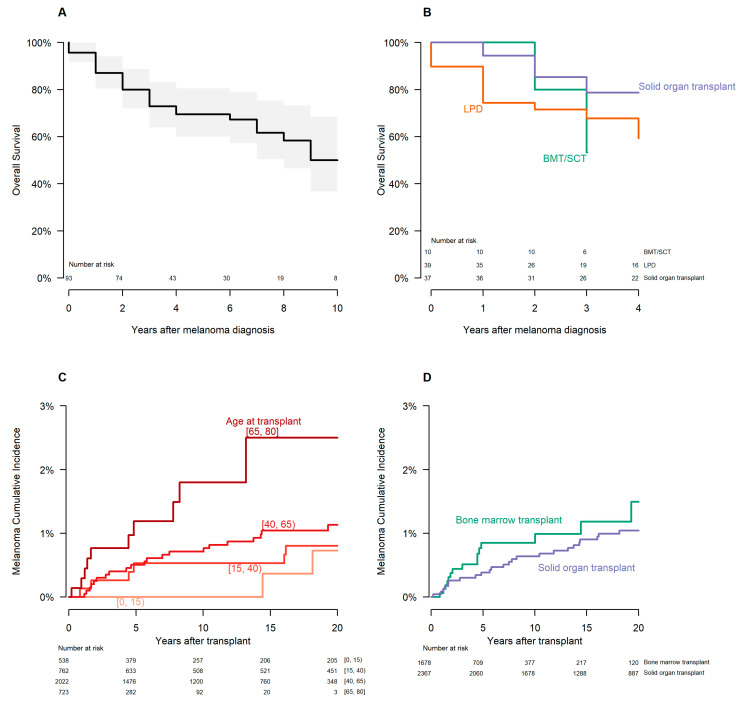
Survival analysis. (**A**): Kaplan–Meier all-cause survival curve of all patients following melanoma diagnosis. The pooled trend is indicated by the solid line, while the 95% confidence interval is indicated by the accompanying gray shading (upper and lower bound). (**B**): Kaplan–Meier all-cause survival curves for immunosuppressive subcategories with n ≥ 10 patients (bone marrow or stem cell transplants, lymphoproliferative disorders, and solid organ transplant). Abbreviations: BMT/SCT, bone marrow transplant/stem cell transplant; LPD, lymphoproliferative disorder. (**C**): Cumulative melanoma incidences for institutional transplant database patients, subdivided by the age of the patient at transplant. Age categories include [0, 15), [15, 40), [40, 65), and [65, 80]. (**D**): Cumulative melanoma incidences for institutional transplant database patients, subdivided by transplant category. Transplant categories include solid organ and bone marrow transplantation.

A survival curve for the entire patient cohort at the time of analysis is shown in Figure 3A. Of the thirty-four deceased patients at the time of analysis, six had a cause of death attributed to melanoma. All-cause survival estimates for all patients were 50% at 10 years after diagnosis (10-year 95% CI 37% to 68%). The median survival estimate was 12 years (interquartile range 3 to 15 years). The median follow-up time among non-deceased patients was 4 years, with a range of 0 to 38 years.

Survival curves for individual immunosuppression subcategories with values of n ≥ 10 were calculated. Subcategories included BMT, LPD, and SOT; survival estimates for these three respective immunosuppressive categories were 53% (95% CI 28% to 100%), 68% (95% CI 54% to 85%), and 79% (95% CI 66% to 94%) at 3 years post-diagnosis. (Figure 3B).

### 3.4. Transplant Sub-Cohort and SEER Analysis

Transplant database records (Table 2) confirmed 9231 bone marrow or solid organ transplants occurring within our 2011–2019 follow-up period, for a total of 51,265 follow-up years and 46 melanoma cases, with an overall melanoma incidence of 0.9 per 1000 years (95% CI 0.66 to 1.20). Melanoma 1000-year incidence rates were highest for autologous BMT (1.98, 95% CI 0.90 to 3.75), followed by allogeneic BMT (1.06, 95% CI 0.43 to 2.19) and finally SOT (0.75, 95% CI 0.50 to 1.07). Consistent with previous literature on the topic [3,4], melanoma 1000-year incidence rates were higher in males (1.12, 95% CI 0.77 to 1.57) than in females (0.60, 95% CI 0.32 to 1.02) (Table 2).

Transplant database patients were divided by age category at the time of transplant and during the follow-up period into the following groups: [0, 15) years old; [15, 40) years old; [40, 65) years old; [65, 80) years old. Cumulative melanoma incidence increased with age, with those in the [65, 80) cohort having the highest incidence per 1000 years at 1.87 (95% CI 1.17 to 2.83) (Figure 3C).

The cumulative incidence of melanoma was similar after BMT or SOT (Figure 3D). The age-adjusted hazard ratio of melanoma for SOT, relative to BMT, was 0.90 (95% CI 0.48 to 1.67). The risk of incident melanoma was higher in the years immediately following the transplant. Since BMT patients averaged less follow-up time in our cohort, the raw incidence of melanoma per follow-up year appears higher in BMT than in SOT (Table 2). However, Figure 3D shows that melanoma risk in any given year of follow-up was much more similar between transplant types.

When compared to the SEER reference population melanoma incidence rates by age at diagnosis of melanoma for the 2011–2019 follow-up period, the overall standardized incidence ratio (SIR) was 1.53 (95% CI 1.12 to 2.04). BMT had an SIR of 3.02 (95% CI 1.73 to 4.90), with allogeneic and autologous BMT with SIR values of 3.18 (95% CI 1.28 to 6.56) and 2.90 (95% CI 1.33 to 5.51), respectively. SOT had an SIR of 1.21 (95% CI 0.82 to 1.73). Males had a standardized incidence ratio of 1.57 (95% CI 1.08 to 2.21), while females had a standardized incidence ratio of 1.43 (95% CI 0.76 to 2.44) (Table 2).

SIRs were also calculated for the same transplant date and follow-up age categories as done for the melanoma incidences above. During the follow-up time, there were no incident melanoma cases among ages 0–14. SIRs were 5.45 (95% CI 2.00 to 11.9) for those aged 15–39, 1.25 (95% CI 0.74 to 1.98) for those aged 40–64, and 1.51 (95% CI 0.94 to 2.28) for those aged 65 and older (Table 2).

## 4. Discussion

While melanoma remains the deadliest form of cutaneous malignancy, immunotherapy has ushered in a new era of innovative and effective treatment for this disease. A better understanding of adaptive immunity and its role in the melanoma microenvironment was critical in the development of these novel therapies. Our study highlights the histopathologic and epidemiologic trends of melanoma in our institution’s cohort of immunocompromised individuals. The most similar study to ours to date is likely Austin et al.’s 2020 cohort study comparing mortality among immunosuppressed patients (SOT, leukemia, lymphoma, and HIV) with melanoma to their non-immunosuppressed peers with melanoma [19]. Other similar published studies include Maor et al.’s report of eleven incident melanoma lesions in a transplant dermatology clinic in Victoria, Australia [20]; Krynitz et al.’s report of melanoma in 49 organ transplant recipients compared to the general population in Sweden [21]; and Park et al.’s report of 51 organ transplant recipients with melanoma and their diagnostic stages and mortality compared to the general population in Ontario, Canada [22]. To our knowledge, our study is one of the largest single-center retrospective cohort analyses of melanoma to include the full spectrum of immunosuppressive disorders. Our research adds to the growing body of literature on this topic by including further categories of immunosuppression (BMT, multiple myeloma, and miscellaneous smaller categories) and comparing a representative patient cohort at an academic tertiary-care center to the SEER database.

Overall, SOT was the most well-represented immunosuppressive subcategory in our patient cohort, particularly kidney transplantation, while LPD and HIV/AIDS were less well-represented. All patients with a leukemia diagnosis had CLL, often a very indolent disease; this has been the most frequently studied leukemia for an association with melanoma development [11]. A 2016 meta-analysis by Olsen et al. found a relative risk ratio of 3.88 for the development of melanoma among those with CLL compared to the general population (95% CI 2.08 to 7.22), with a greater risk noted for men with CLL compared to women [23]. Another study by Brewer et al. found a 2.6-fold worse survival and a 2.8-fold higher likelihood of dying from metastatic melanoma among patients who also had a history of CLL compared to those with melanoma alone [24]. A 2022 study by Jobson et al., published in the British Journal of Haematology, examined a retrospective cohort of 56 melanoma patients with a history of CLL and matched them to 56 control melanoma patients without a prior CLL history. The investigators found significantly higher rates of melanoma-specific mortality and lower rates of recurrence-free survival among those with a CLL history. Interestingly, the authors found no association between CLL treatment and an impact on melanoma mortality and recurrence; they hypothesized that a history of immunosuppressive regimen and lymphocyte depletion would be unlikely to affect the outcome of melanoma diagnosed potentially many years later [25].

The association between HIV/AIDS and melanoma development is less well-established than SOT and LPDs. A 2014 meta-analysis by Olsen et al. found an overall post-HAART era relative risk of individuals with HIV/AIDS developing melanoma of 1.26 (95% CI 0.97 to 1.64) and, when adjusted for ethnicity, 1.50 (95% CI 1.12 to 2.01). The researchers noted significant heterogeneity among the included studies, and confounding factors may play a role in melanoma development in this patient demographic [15,26]. A systematic review with the inclusion of studies until 2012 found that, among non-AIDS-defining cancers (as opposed to AIDS-defining cancers), there was no statistically significant increase or decrease in rates of melanoma between the pre- and post-HAART era. In contrast, rates of anal, colorectal, Hodgkin lymphoma, liver, lung, and prostate cancers increased after the introduction of HAART. Although it was unclear why the incidence of some cancers increased after the introduction of HAART while others did not, the authors suggest that the genotoxic effects of zidovudine and increased life expectancy among HIV/AIDS patients may lead to a longer time for some forms of cancer to develop [27]. A 2022 meta-analysis conducted by Yuan et al. found that individuals with HIV/AIDS were more likely to die from five non-AIDS-defining cancers than their non-infected peers, including anal cancer, Hodgkin lymphoma, liver cancer, lung cancer, and cutaneous melanoma [28]. A comprehensive analysis of HIV and non-keratinocyte skin cancers did not see a statistically significant increase in melanoma in HIV patients compared to the general population; the authors even suggested that in patients with HIV lacking other significant skin cancer risk factors, intense skin surveillance may be unnecessary [29].

We did not observe large differences in Breslow depth or Clark level across immunosuppressive categories, though it is difficult to make a final determination on whether variation exists between groups due to the relatively small sample sizes in each category and the lack of full histopathologic data in some patients. Previous investigators have also expressed some difficulty due to this issue, for example, in the Brewer et al., 2011 study comparing mortality among SOT recipients at the Mayo Clinic and other sites to the SEER database; in their research, Breslow thickness was available for 123 of 638 post-transplant melanoma patients and Clark level for 175 of 638 post-transplant melanoma patients [30]. Maor et al.’s report of eleven incident melanomas in a Victoria, Australia transplant clinic had a benefit in this regard of having the full melanoma subtype, Breslow depth, and Clark level available for their patients [20]. In our study, it is also difficult to determine whether the median time from immunosuppressive onset to melanoma diagnosis varies significantly across immunosuppressive categories, given the differential clinical follow-up time between groups. Kaplan–Meier survival estimates suggested a lower overall survival rate for patients with BMT compared with SOT and LPD; however, this difference was not enough to reject the null hypothesis of no statistically significant difference between these groups. Likewise, there was no statistically significant difference in survival estimates observed between melanoma patients with a history of SOT and LPD. Overall, there appeared to be no statistically significant differences in histopathologic or survival data for melanoma patients based on the cause of immunosuppression. This may indicate that factors other than the immunosuppressive category may be more predictive of melanoma histopathology and survival. For instance, potentially confounding variables may include age at diagnosis, sex, comorbidities, immunosuppressive regimens for transplant patients, and previous history of other skin cancers.

The overall incidence of melanoma in the transplant population was 0.9 per 1000 person-years, though this varied by age, sex, and time since transplantation. The incidence of melanoma per 1000 person-years increased in each successive age cohort during the follow-up period. Additionally, the melanoma incidence appeared to rise most dramatically in the few years immediately after transplantation. For example, in Figure 3D, melanoma incidence increased as much from years 0 to 5 post-transplantation as it did from years 5 to 20 post-transplantation, especially in BMT patients.

The overall melanoma SIR for our transplant cohort was 1.53 (95% CI 1.12 to 2.04), though this varied by transplant type, sex, and time since transplantation. Consistent with previous findings [3,4], males had approximately twice the risk of developing melanoma as females, though SIRs between the two groups were very similar. Although differences were noted between BMT and SOT patients in terms of melanoma incidence and SIRs, this is likely explained by the fact that BMT patients had much higher mortality in the post-transplant period and represented a much lower percentage of all transplants (compared to SOT) prior to 2010. Cumulative incidences (see Figure 3D) and hazard ratios would suggest that BMT and SOT patients have, in fact, a similar risk of developing melanoma at any given time relative to the transplant. In comparison, previous systematic reviews have suggested relative risks of melanoma development among solid organ transplant recipients of 2.71 (95% CI 2.23 to 3.30) and 2.4 (95% CI 2.0 to 2.9) among 20 and 12 individual studies, respectively; variation in relative risk was observed based on the type of organ transplanted in these reviews [31,32]. It is noteworthy that our study’s SIR for SOT included the null value, suggesting no significant difference in rates of melanoma development between our SOT sub-cohort and the general population, in contrast to the aforementioned systematic reviews. In addition, the mean follow-up time for our SOT cohort was almost 10 years, and the SIR was calculated over all years of follow-up, with melanoma incidence being the highest in the years immediately following the transplant. This relatively long follow-up time could explain the discrepancy between our study and prior literature. Moreover, it is important to note that significant study heterogeneity, as seen in the 2015 Green and Olsen meta-analysis, has been observed [31]. We hypothesize that a variety of confounding factors, including changes in immunosuppressive regimens over the decades, a better understanding of the relationship between immunosuppression and melanoma, and more vigilant clinical surveillance, may help to account for the heterogeneity of studies on SOT and melanoma to date. The type of immunosuppressive regimen, dose, and duration are also likely to be significant confounding variables. Other researchers have noted this limitation in studying the effects of immunosuppressive therapies on melanoma development and noted that it is likely very difficult to directly compare the effects of widely varying therapies between individual patients [22,33], an undertaking we did not directly investigate in our analysis.

There is a discrepancy in the literature to date regarding the effect of SOT and associated immunosuppression on the overall survival and prognosis of melanoma [11]. Overall, most studies have found that immunosuppression associated with SOT worsens 2-, 5-, and 10-year survival rates for melanoma; the most comprehensive retrospective cohort study by Brewer et al. using SEER data found significantly worse survival rates among transplant recipients compared to the general population, regardless of Breslow depth or Clark level [11,30,32]. Moreover, the aforementioned relatively recent international cohort studies have suggested that melanoma lesions in transplant recipients, when compared to the general population, are more advanced at diagnosis and carry a higher risk of melanoma-specific death [21,22]. Maor et al.’s cohort study at a dedicated transplant dermatology clinic in Victoria, Australia, found a “needed to excise” pigmented lesion ratio of 16:1 (i.e., 16 pigmented lesions required excision to diagnose melanoma in this patient population); apart from one heart transplant recipient, all patients in the cohort had undergone renal transplantation [20]. Although our study did report trends in overall survival for the entire patient cohort (Figure 3A) and for well-represented subcategories of immunosuppression (Figure 3B), we did not specifically compare the overall survival of our cohort to the overall survival of the SEER population following melanoma diagnosis. The SEER database provided survival relative to an age and sex-matched US population, which was 90% at 10 years; in other words, the proportion of SEER melanoma 10-year survivors was 90% that of the US population with the same age and sex distribution. We also computed relative survival for our study cohort versus the US population using the 2018 US Life Table from the CDC [34]. For the US, an age and sex-matched cohort of non-Hispanic whites had expected ten-year overall survival of 74%. Ten-year overall survival in our cohort was 50%. Thus, the ten-year relative survival for our cohort was 68% (50/74), which was considerably lower than the 90% relative survival for the SEER melanoma cohort. We also did not complete a full analysis of melanoma-specific survival compared with the SEER population; overall, 6 of the 34 deceased patients (17.6%) had a cause of death attributed to melanoma. Studies that have analyzed melanoma-specific survival include Austin et al.’s 2020 cohort study, which found that immunosuppressed patients (SOT, leukemia, lymphoma, and HIV) with melanoma had a higher incidence of non-melanoma deaths but a similar incidence of melanoma deaths compared to non-immunosuppressed patients [19]. The authors suggested that increased incidences of death from other causes post-transplant might paradoxically reduce melanoma deaths [19]. In contrast, Park et al.’s 2020 cohort study in Ontario found lower melanoma-specific survival among solid organ transplant patients compared to their non-transplant peers [22]. Further studies involving multiple subcategories of immunosuppression are likely needed to fully elucidate the effect of immunosuppression on melanoma-specific survival. Regardless, close clinical follow-up and coordinated care are essential in the transplant patient demographic to monitor for the development of cutaneous malignancy, including melanoma.

In our study, age at transplant remained a confounder between allogeneic and autologous BMT recipients in terms of raw incidence of melanoma but was controlled for in our SIR analysis. The initial analysis of melanoma incidence suggested that autologous patients had higher rates than allogeneic patients (1.98 per 1000 person-years versus 1.06 per 1000 person-years, respectively). When age-adjusted by SEER data, allogeneic and autologous BMT SIRs were both near three (3.18 and 2.90, respectively). It appears overall that the type of BMT does not play a major role in melanoma incidence when adjusted for age as the major confounding variable between allogeneic and autologous groups.

As expected, melanoma incidence increased with increasing age at follow-up, especially in patients over 65 years old. Interestingly, individuals aged 15–39 appeared to have an SIR significantly higher than other age cohorts (5.45), though, in absolute terms, there were still only six cases of melanoma reported during the follow-up period in this age cohort versus 1.1 cases expected. Note that no cases of melanoma were diagnosed in individuals aged 0–14 years old during the follow-up period. The overall finding of increased risk of melanoma incidence with older age is consistent with previous data and likely attributed to a variety of factors, including increased lifetime exposure to UV radiation, increased immune senescence, and acquisition of other comorbidities for melanoma development.

Like the BMT subtype, the SOT subtype did not appear to significantly affect the overall incidence or SIR of melanoma. Although liver transplant patients had a higher hazard ratio (1.87) and SIR (2.0) than other transplant subtypes, the 95% confidence intervals included the null value (1). It is likely that other factors, including sex and age during the follow-up period, play a much larger role in melanoma incidence for these solid organ transplant patients.

Interestingly, the most represented melanoma location (data available for 107 of 111 melanoma lesions) was the head and neck region (n = 43 lesions). When subdivided by sex, males accounted for the majority of these cases, even when adjusted for the proportion of the total cohort (n = 36 lesions). In studies similar to ours, the trunk has generally been found to be the most commonly involved site [21,35], although some studies have noted a preponderance on the head, neck, and upper extremities and have recognized this discordance between their studies and the literature overall [20,36].

It is also interesting to draw a parallel with other studies, where malignant melanoma patients with a history of transplantation have shown worse overall survival and disease-free survival [36,37], especially when the patients developed more advanced disease compared with a control population [35].

Strengths of our study include the detailed data collection, including survival data, enabling a comprehensive data analysis; the relatively large number of patients for a study of its kind using data from a single institution; the wide range of immunosuppressive backgrounds of the cohort, allowing for subgroup analysis and a sense of how widely-represented each subcategory of immunosuppression might be at a single institution; the available detailed information about the entire transplant population at our institution, and the inclusion of both SOT and BMT; relatively straightforward comparability to prior studies given the similar methodology and clinical and histopathologic data that we collected; and the overall methodology allowing for easy comparison to the SEER population. Prior studies [10,19,24,30,35,38,39,40,41,42,43] have investigated the effect of either one particular (e.g., SOT) or multiple causes of immunosuppression on melanoma incidence and prognosis; our study adds significantly to this body of literature by including and analyzing several immunosuppressive conditions in a single-center cohort of melanoma patients and then comparing that cohort with the general population found in the SEER database.

A retrospective cohort analysis such as ours is likely subject to some degree of surveillance bias. Specifically, patients with an immunosuppressive history are more likely to be receiving frequent follow-ups, specialist visits, and skin checks that could lead to greater detection of melanoma lesions and precursor lesions than the general public. Another limitation of our study was the fact that for some lesions, we were not able to retrieve important clinical and pathologic data, although this occurred for a relatively small number of variables, most notably Breslow depth and Clark level. An inherent limitation to our and similar studies is the fact that it is essentially impossible to ascertain precisely when a melanoma develops (pre- vs. post-onset of immunosuppression) from a dysplastic precursor lesion. For this reason, it would not be appropriate to attribute a case of melanoma solely to immunosuppression without considering other patient risk factors such as genetic mutations, sex, sun exposure, and sun protective behaviors, among others. Although organ donors are extensively screened for malignancy prior to transplantation, it is also not impossible for donor-to-recipient transmission of melanoma to occur [44], a point that other investigators have also recognized [22,33]. We did not include data regarding prior history of dysplastic nevi, non-melanoma skin cancer, or specific details on immunosuppressive regimens (when applicable), which may have facilitated a further dimension of analysis in our study (although beyond the scope of our aims). Finally, the sample size for some subgroups was small, and all the cases were from a single institution, thus limiting the generalization of some findings. Ideally, a larger sample size and pooling of cases from multiple institutions may refine the assessment of risk in this population of immunocompromised patients.

## 5. Conclusions

This study investigated the full available clinical and histopathologic data in a cohort of melanoma patients with a prior history of immunosuppression from our institution, a large academic tertiary-care center. In our cohort, transplantation, particularly solid organ transplantation, was the most common cause of preceding immunosuppression in such a patient group, with an increased risk of melanoma incidence in male patients. Our results revealed no statistically significant differences in Kaplan–Meier all-cause survival estimates between transplant subtypes and/or lymphoproliferative disorders. The overall incidence of melanoma in our transplant sub-cohort was 0.9 per 1000 person-years with a standardized incidence ratio of 1.53, with variation noted based on transplant type, sex, and time since transplantation. Melanoma incidences rose in relation to patient age during the follow-up period; patients over age 65 had the highest incidence, while those in the age range of 15–39 at diagnosis had the highest SIR relative to the general population.

Surveillance detection bias is a potential confounding variable in this type of retrospective cohort analysis, as immunosuppressed patients are more likely to be receiving regular follow-up medical care and skin examinations than their non-immunosuppressed counterparts.

Our study offers a perspective on melanoma in immunocompromised patients at a university-based tertiary care center and connected clinics.

## Figures and Tables

**Table 1:** Basic patient demographic and lesion histopathologic data. Overall patient cohort demographic characteristics, including gender (n = 93 patients), vital status (n = 93 patients), cause of immunosuppression (n = 93 patients), lesion location (n = 107 lesions), melanoma stage (n = 101 lesions), melanoma subtype (n = 57 lesions), melanoma primary tumor classification (n = 41 lesions), and time from immunosuppressive onset to melanoma diagnosis (n = 93 patients).

Descriptive Clinical and Pathologic Data	
Gender (n = 93)	
Male	65
Female	28
All-cause mortality (n = 93)	
Alive	59
Deceased	34
Causes of immunosuppression (n = 93)	
Solid organ transplant	37
Kidney	20
Liver	9
Kidney and pancreas simultaneously	4
Heart	2
Lung	2
Lymphoma	23
Bone marrow/stem cell transplant	10
Leukemia	9
Multiple myeloma	6
HIV/AIDS	4
Chronic granulomatous disease	1
Hypogammaglobulinemia	1
Immunoglobulin deficiency	1
Monoclonal gammopathy of undetermined significance	1
Lesion location (n = 107)	
Head and neck	43
Upper extremity	28
Trunk	27
Lower extremity	7
Eye	2
Melanoma stage (n = 101)	
Invasive	68
In situ	33
Melanoma subtype (n = 57)	
Superficial spreading melanoma	17
Lentigo maligna melanoma	13
Melanoma in situ, lentigo maligna type	13
Nodular melanoma	5
Melanoma, not otherwise classified	3
Metastatic melanoma of unknown primary	3
Acral melanoma	1
Amelanotic melanoma	1
Melanoma in situ arising within a compound nevus	1
Melanoma primary tumor classification (n = 41)	
Tis	2
T1a	20
T1b	7
T2a	6
T2b	3
T3a	1
T3b	1
T4b	1
Years from immunosuppression onset to initial melanoma diagnosis, median (min-max)	
All cases (n = 93)	5 (0–47)
All transplants (n = 47)	7 (0–47)
Solid organ transplant (n = 37)	7 (0–47)
Bone marrow/stem cell transplant (n = 10)	4 (0–24)
Lymphoproliferative disorder (n = 39)	4 (0–26)

**Table 2:** Institutional transplant database and SEER analysis. Melanoma incidence in our institutional transplant database for white, non-Hispanic patients compared with SEER epidemiologic data. “SIR” (standardized incidence ratio) was calculated using the SEER-21 registry as a reference population, adjusting for age and sex and including only non-Hispanic whites in both the study and reference populations (all patients in our transplant cohort with a recorded racial/ethnic group were non-Hispanic white). For age, “N” represents the number of patients who have full or partial follow-up time in each respective category. Patients may age into a new category during follow-up. “Mean years since tx” is defined as the years between the transplant and the midpoint of each patient’s follow-up time. Note that melanoma incidence is higher in the years immediately following transplant, so the higher incidence and SIR for BMT compared to solid organ transplant is partially due to the lower mean time elapsed since transplant for BMT. Patients with a history of autologous BMT were included in the SEER analysis even though only allogeneic BMT was considered a primary cause of immunosuppression as in Table 1; in other words, we ascribed immunosuppression to other causes, generally relating to lymphoproliferative disorders, for patients with an autologous BMT history. Abbreviations: mean years since tx, years between transplant, and the midpoint of each patient’s follow-up time; 95% CI, 95% confidence interval.

Subgroup	N	Melanoma Cases	Total Years of Follow-Up	Mean Years Since tx	Incidence per 1000 Years	95% CI	SIR	95% CI
Overall	9231	46	51,265	8.3	0.90	(0.66, 1.20)	1.53	(1.12, 2.04)
Transplant type								
Solid organ	6535	30	40,117	9.7	0.75	(0.50, 1.07)	1.21	(0.82, 1.73)
BMT	2696	16	11,148	5.0	1.44	(0.82, 2.33)	3.02	(1.73, 4.90)
Allogeneic	1571	7	6594	5.6	1.06	(0.43, 2.19)	3.18	(1.28, 6.56)
Autologous	1125	9	4554	4.2	1.98	(0.90, 3.75)	2.90	(1.33, 5.51)
Sex								
Female	3840	13	21,754	8.4	0.60	(0.32, 1.02)	1.43	(0.76, 2.44)
Male	5391	33	29,511	8.3	1.12	(0.77, 1.57)	1.57	(1.08, 2.21)
Transplant year								
1964–1990	596	4	4239	29.3	0.94	(0.26, 2.42)	1.48	(0.40, 3.79)
1991–2000	1553	11	10,986	17.8	1.00	(0.50, 1.79)	1.69	(0.84, 3.03)
2001–2010	3075	9	21,476	8.0	0.42	(0.19, 0.80)	0.70	(0.32, 1.32)
2011–2015	2304	15	11,389	2.5	1.32	(0.74, 2.17)	2.46	(1.38, 4.06)
2016–2019	1709	7	3175	0.9	2.20	(0.89, 4.54)	3.89	(1.56, 8.01)
Age during follow-up								
0–14	877	0	3178	3.7	0.00	(0.00, 1.16)		
15–39	2141	6	10,141	8.4	0.59	(0.22, 1.29)	5.45	(2.00, 11.9)
40–64	5458	18	26,156	8.9	0.69	(0.41, 1.09)	1.25	(0.74, 1.98)
65 and older	2926	22	11,786	9.9	1.87	(1.17, 2.83)	1.51	(0.94, 2.28)
Time since transplant								
Less than 5 years	5790	21	16,375	2.2	1.28	(0.79, 1.96)	2.41	(1.49, 3.69)
More than 5 years	6212	25	34,890	11.0	0.72	(0.46, 1.06)	1.21	(0.78, 1.78)

## Data Availability

Data are available upon request.
